# A randomized matched-pairs study of feasibility, acceptability, and effectiveness of systems consultation: a novel implementation strategy for adopting clinical guidelines for Opioid prescribing in primary care

**DOI:** 10.1186/s13012-018-0713-1

**Published:** 2018-01-25

**Authors:** Andrew Quanbeck, Randall T. Brown, Aleksandra E. Zgierska, Nora Jacobson, James M. Robinson, Roberta A. Johnson, Brienna M. Deyo, Lynn Madden, Wen-Jan Tuan, Esra Alagoz

**Affiliations:** 1 University of Wisconsin Department of Family Medicine and Community Health, Madison, WI USA; 2 University of Wisconsin Center for Health Enhancement Systems Studies, Madison, WI USA; 3 University of Wisconsin – Madison School of Nursing, Madison, WI USA; 4 University of Wisconsin Center for Health Systems Research and Analysis, Madison, WI USA; 5 APT Foundation, New Haven, CT USA

**Keywords:** Opioid prescribing, Evidence-based practice, Organizational coaching, Clinical practice guidelines, Organizational implementation strategies, Primary care

## Abstract

**Background:**

This paper reports on the feasibility, acceptability, and effectiveness of an innovative implementation strategy named “systems consultation” aimed at improving adherence to clinical guidelines for opioid prescribing in primary care. While clinical guidelines for opioid prescribing have been developed, they have not been widely implemented, even as opioid abuse reaches epidemic levels.

**Methods:**

We tested a blended implementation strategy consisting of several discrete implementation strategies, including audit and feedback, academic detailing, and external facilitation. The study compares four intervention clinics to four control clinics in a randomized matched-pairs design. Each systems consultant aided clinics on implementing the guidelines during a 6-month intervention consisting of monthly site visits and teleconferences/videoconferences. The mixed-methods evaluation employs the RE-AIM (Reach, Effectiveness, Adoption, Implementation, Maintenance) framework. Quantitative outcomes are compared using time series analysis. Qualitative methods included focus groups, structured interviews, and ethnographic field techniques.

**Results:**

Seven clinics were randomly approached to recruit four intervention clinics. Each clinic designated a project team consisting of six to eight staff members, each with at least one prescriber. Attendance at intervention meetings was 83%. More than 80% of staff respondents agreed or strongly agreed with the statements: “I am more familiar with guidelines for safe opioid prescribing” and “My clinic’s workflow for opioid prescribing is easier.” At 6 months, statistically significant improvements were noted in intervention clinics in the percentage of patients with mental health screens, treatment agreements, urine drug tests, and opioid-benzodiazepine co-prescribing. At 12 months, morphine-equivalent daily dose was significantly reduced in intervention clinics compared to controls. The cost to deliver the strategy was $7345 per clinic. Adaptations were required to make the strategy more acceptable for primary care. Qualitatively, intervention clinics reported that chronic pain was now treated using approaches similar to those employed for other chronic conditions, such as hypertension and diabetes.

**Conclusions:**

The systems consultation implementation strategy demonstrated feasibility, acceptability, and effectiveness in a study involving eight primary care clinics. This multi-disciplinary strategy holds potential to mitigate the prevalence of opioid addiction and ultimately may help to improve implementation of clinical guidelines across healthcare.

**Trial registration:**

ClinicalTrials.gov (NCT02433496). https://clinicaltrials.gov/ct2/show/NCT02433496

Registered May 5, 2015

**Electronic supplementary material:**

The online version of this article (10.1186/s13012-018-0713-1) contains supplementary material, which is available to authorized users.

## Background

### Implementing clinical guidelines in healthcare organizations

Healthcare adopts evidence-based practices (EBPs) notoriously slowly [1]. Traditional approaches to improving medical practice have relied upon experts producing and publishing clinical guidelines in academic journals. Various methods have been tried to help clinics adopt clinical guidelines and other EBPs, such as providing educational materials, audit/feedback [2], and academic detailing [3], with mixed results. Despite the use of such methods, only about 55% of adults in the USA receive recommended care for 30 acute and chronic conditions [4], a rate that has remained relatively stable since it was first reported in 2003 [5]. Many of the chronic conditions reported in these studies—such as hypertension, diabetes, asthma, and hypertension—are usually treated in primary care, the setting of interest in this paper.

Much has been published about the problem of clinical guideline uptake and possible solutions to it [6–10]. Various explanations of the problem and possible solutions have been identified—e.g., relating the process of developing guidelines to their uptake, or changing the methods used to disseminate the guidelines, or determining how implementable the guidelines are. The best approaches to bridging the gap between medical research and clinical practice are not yet known.

### Opioid prescribing

Increases in prescription opioids misuse have raised alarms throughout the USA [11]. The problem is now spreading to Canada and other countries [12]. Since 1999, the number of opioid overdose deaths in the USA has quadrupled, as has the amount of prescription opioids dispensed [13]. Prescription opioids account for more than half of overdose deaths [14], and about half of opioid prescriptions are written in primary care [15]. Prescribing opioids for chronic non-cancer pain is accompanied by a dose-dependent risk of addiction and overdose [16–18]. Additionally, patients at increased risk for misuse (i.e., those with mental health and/or substance use disorders) are more likely to receive opioid prescriptions and higher daily doses [16, 19–21]. By the end of 2016, seven states had adopted laws limiting opioid prescribing [22]. Federally, various agencies (the Substance Abuse and Mental Health Services Administration, the Centers for Disease Control and Prevention, and the National Institutes of Health) have issued new guidelines and approaches to opioid prescribing [23].

The prescription opioids crisis requires an effort to de-adopt potentially harmful clinical practices. Evidence-based clinical guidelines have been developed for opioid prescribing. They advocate such procedures as screening for mental health and substance abuse issues, using treatment agreements (which document risks and safeguards related to taking opioids and are signed by prescribers and patients), and urine drug testing. Nonetheless, the uptake of these guidelines varies among primary care clinics [24]. Information about safe opioid use also has been published for patients [25], but treatment agreements and conversations with prescribers are the most common way that patients receive information about safe opioid use.

### The implementation strategy

This study reports on a novel implementation strategy, called systems consultation, designed to promote clinical guideline implementation for opioid prescribing in primary care. The term “systems consultation” was inspired by an article entitled “Controlling variation in healthcare: a consultation from Walter Shewhart” [26]. Shewhart was an iconic systems engineer; the name “systems consultation” refers to the strategy’s roots in systems engineering.

Systems consultation arose from a study that tested various approaches to making organizational change in one of the largest cluster-randomized quality improvement (QI) trials conducted in US healthcare [27]. Among the tested approaches, organizational coaching—a critical element of systems consultation—proved to be the most cost-effective in that study of 201 addiction treatment clinics [27]. (The term *coaching* was changed to *consulting* in this study because we heard from primary care physicians that “doctors don’t like to be coached.”) Systems consultation is a blended implementation strategy offering a bundle of integrated approaches to improve the uptake of EBPs [28, 29], including (1) audit and feedback, which consists of providing performance feedback to clinics that serves as baseline information and points to opportunities for improvement; (2) academic detailing, in which a respected physician with expertise in addiction medicine visits clinics to provide advice on how to improve clinical practice; and (3) organizational coaching (or *external facilitation*, the term common in primary care), an intensive advising approach designed to tailor guideline or policy recommendations to specific clinical contexts.

Blended approaches have been suggested to speed the implementation of evidence-based practices in general [30, 31] and the implementation of clinical guidelines specifically [32]. The particular blend of strategies in the systems consultation model has not been tested before, although evidence exists for each of its elements [27, 33, 34].

We initially expected the physician consultants who provided academic detailing also to take on the more intensive external facilitation role, but it became clear that they needed more support to do this and that facilitation could be capably handled by another research team member with less training (i.e., master’s level). As a result, the physician consultant and the other research team member, the facilitator, formed a “consultation team.” The facilitator provided data and other support to clinics, arranged meetings, went on regular site visits with the physician consultant, and led the clinic’s use of tools from systems engineering (e.g., walkthrough exercises [35], flowcharting [35], nominal group technique [36], and Plan-Do-Study-Act (PDSA) cycles [37]) to help clinics make improvements in clinic processes. This team structure mirrors the structure of care teams in most primary care clinics, in that the consultation team was led by a physician who delegated more time-intensive activities to a staff member with less education and training.

### Study aim

Systems consultation was rated by NIH grant reviewers as innovative but untested, and thus suitable for NIH’s R34 clinical trial planning grant mechanism. The aim of the study reported in this paper was to pilot test the systems consultation strategy in a small set of primary care clinics to see if the strategy demonstrated feasibility, acceptability, and preliminary effectiveness in improving clinician adherence to opioid-prescribing guidelines and reducing morphine-equivalent daily dose (MEDD) for patients on long-term opioid therapy. If so, we would plan a follow-up study to test the approach in a large-scale randomized trial. If ultimately proven effective, systems consultation could be applied on a population level to help combat the prescription opioids crisis and possibly be applied to other clinical guidelines.

## Methods

### Developing the content of the implementation strategy

Developing clinical guidelines typically involves panels of experts systematically reviewing the literature, discussing the results in light of their expertise and experience, achieving consensus, and publishing the results in a journal intended for the clinical audience. This approach has left a substantial gap between clinical knowledge and clinical practice [38]. Systems consultation adds a novel step in promoting guideline uptake by bringing together guideline writers, implementation experts, and primary care physicians to translate clinical guidelines into a succinct, checklist-based format that can be readily implemented. For this project, the experts convened included physicians from the panel that developed one of the leading opioid prescribing guidelines [24]; internationally recognized experts on implementation research, healthcare QI, and drug policy; and community-based family medicine physicians (see the “Acknowledgements” section). We followed a systematic group decision-making approach called the integrative group process [39], a set of techniques for facilitating meetings of experts. We began by conducting a structured Delphi process [36] in which we asked each member of the advisory panel to rate each recommendation in the opioid prescribing clinical guideline on its measurability, potential to reduce opioid abuse, and ease of implementation. Principal investigators Quanbeck and Brown then conducted follow-up telephone interviews with each of the nine panel members to understand the ratings they assigned. Quanbeck and Brown synthesized their notes from these interviews into a preliminary checklist. This checklist was the subject of a full-day, in-person meeting of the entire advisory panel held in November 2014. During the meeting, the research team presented the initial checklist and asked panel members to provide feedback and revisions. We presented a variety of archetypal patient cases (e.g., a new chronic pain patient being considered for opioid therapy versus a patient already established on long-term opioids) and asked panel members to discuss how the checklist should be adapted to fit different circumstances and local contexts (which research suggests is vital to successful implementation) [40].

Teaming panelists who wrote the clinical guideline for opioid prescribing [24] with implementation experts and primary care physicians produced an implementation guide that formed the content of the intervention. This guide consisted of a checklist of the essential elements of the clinical guidelines and a set of systems engineering tools designed to assist with local customization of the implementation strategy. The checklist initially specified a target dose limit of 120 MEDD. When CDC guidelines were issued later, this target was lowered to 90 MEDD to agree with the CDC standard (although we used 120 MEDD for evaluation). Developing the implementation guide also clarified elements of the implementation. For example, the question of where to start the work in a clinic, which could have been daunting, was answered by initially focusing on the opioid prescription refill process for existing patients. Each clinic has a process for prescription refills, and refill requests are relatively predictable—whereas the arrival of a new patient with a chronic pain complaint is not. The goal of the improvement effort was framed as using the checklist to put safeguards in place for all patients, beginning with patients already on long-term opioid therapy.

### Implementation study design

The study employed a randomized matched-pair design conducted with eight community-based primary care clinics, with four clinics receiving the intervention and four clinics serving as controls. Given the novelty of the implementation strategy, we decided to study a small number of clinics in detail, using both quantitative and qualitative methods, rather than powering the study as a randomized trial of effectiveness. During the 6-month intervention, physician consultants met with small groups called “change teams” at each intervention clinic at months 1, 2, and 6 and had teleconferences or videoconferences with change teams in months 3, 4, and 5. Occasional email and phone correspondence took place between physician consultants and clinic staff; more frequent email correspondence took place between the study facilitator and change team members.

### Setting and ethics approval

The study took place in family medicine clinics that are part of UWHealth, the health system affiliated with the University of Wisconsin Department of Family Medicine and Community Health. The intervention was introduced to the four intervention clinics on staggered starting dates between February and May 2016, ending in each clinic 6 months later. The study protocol was reviewed and approved by the University of Wisconsin – Madison’s Health Sciences Institutional Review Board, submission 2015-0280-CR002.

### Study participants

#### Consultation team

The two physician consultants are faculty members in the University of Wisconsin’s Department of Family Medicine and Community Health. Both are board-certified in family medicine and addiction medicine and have current clinical practices in the same health system as participating clinics. They have extensive clinical experience with opioid therapy in accordance with clinical guidelines. Each consultant worked with two intervention clinics. The consultants received training from two experienced organizational coaches [41] before the intervention began and ongoing advice during the intervention. The training covered systems engineering principles and tools such as using the walk-through exercise, flowcharting, nominal group technique, and Plan-Do-Study-Act cycles [35]. The study facilitator scheduled meetings, prepared materials, kept records, helped clinics use systems engineering tools, and answered questions from the clinic change team participants during the intervention.

#### Clinics and change teams

Clinic characteristics are shown in Table 1. Each clinic was asked to designate a change team of six to eight staff members to work on the project. Each change team had at least one prescribing clinician, one registered nurse, one medical assistant or licensed practical nurse, and an administrative staff member, such as a receptionist. Each change team had a leader who either volunteered for or was nominated by the team for the role. The leader was responsible for communicating with the research team.

**Table 1:** Clinic and patient characteristics

Clinics	Intervention (*n* = 4)	Controls (*n* = 4)	Refused (*n* = 3)
Average number of prescribers (MD, PA, NP)	8.5	3.3	6.7
Average number annual patients	7489	3324	3271
% Female	47.1	49.7	45.6
% Hispanic	2.2	2.5	2.5
% Asian	2.4	1.6	0.7
% Black	2.4	2.3	0.7
% Native	0.5	0.5	0.5
% Other	11.9	12.2	11.5
% White	82.8	83.6	86.7

### Study procedures

#### Clinic recruitment and randomization

**Fig. 1:**
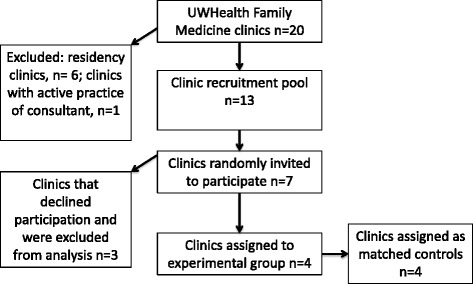
Clinic recruitment flow diagram

Recruitment in this unblinded trial focused on the 20 family medicine clinics in the UWHealth system. Clinics offering resident training were excluded (*n* = 6); one clinic was excluded because one of the physician consultants had an active practice there. We selected the remaining 13 clinics as a recruitment pool to enable systematic monitoring through the clinics’ common electronic health record. The 13 clinics were first grouped into two categories (urban vs. rural) and then ranked from largest to smallest by the number of patients with consistent opioid prescriptions (defined as three or more opioid prescriptions in each of the most recent 3 months). Then, we selected the three largest of the four pairs in the urban group and the larger of the two pairs in the rural group. Within each pairing, one clinic was invited to be the intervention clinic by random selection using a computerized random number generator. If that clinic agreed to participate, the second clinic in the pairing was assigned to the control condition. If the first clinic declined to participate, the second clinic in the pair was invited to be the intervention clinic. If the second clinic agreed to participate, it was assigned to be the intervention clinic, and the closest matching clinic from the remainder of the recruitment pool was assigned to the control condition (Fig. 1).

Recruitment began with obtaining permission to conduct the study and recruit clinics from the UWHealth Primary Care Leadership Council. Then, in January 2016, the two physician consultants wrote emails to the medical directors of clinics that had been randomly selected from each pairing. The emails included a one-page flyer about the study. The first three clinics declined to participate, citing a lack of time or turnover of key staff (clinic managers or medical directors). The next four clinics that were approached agreed to participate, with the final clinic agreeing to participate in April 2016. In summary, we randomly approached seven clinics to recruit four. The intervention clinics consisted of four of the eight clinics in the initial selection pool; the control clinics consisted of one clinic from the initial selection pool and three from outside the original selection pool.

#### Implementation timeline

Before the 6-month intervention period began, consultants conducted informational meetings at the clinics to discuss the opioid prescribing problem and the goals of the study. After this, the study facilitator went to each clinic to complete the informed consent process and conduct a walk-through of the opioid prescription refill process. In the walk-through, the facilitator and change team members followed the steps in the process of refilling an opioid prescription, starting with the patient calling or coming to the clinic and ending with the patient having the refill in hand. This process was summarized in a flowchart designed to show how the process worked, and in so doing, reveal inefficiencies, redundancies, and opportunities for improvement.

After the walkthrough and flowchart were done, the intervention officially began with the first of six monthly meetings between the consulting team and clinic change teams. The consulting teams visited the clinics for these meetings in months 1, 2, and 6; in months 3, 4, and 5, the meetings took place via videoconference or teleconference. Lunches were supplied to the change team and the research team members during the on-site meetings, which lasted 1 hour and took place during lunchtime. At the suggestion of the intervention clinics, a telephone conference involving all four clinics took place at the end of July 2016 (roughly the middle of each clinic’s intervention) to give the change teams a chance to learn from one another. Each team reported on its work, results, and what they had learned to date. Researchers who attended the meetings were, in addition to the physician consultants, the study facilitator, an organizational coach from systems engineering, and a qualitative researcher.

During the first meeting with clinic change teams, the physician consultant introduced two systems engineering tools, the nominal group technique and PDSA cycles. The nominal group technique [36] is a method for group decision-making used to identify problems or solutions. It begins with the meeting organizer posing a question, such as, “What problem in our opioid prescribing process is most important to solve first?” Change team members individually brainstorm answers to this question, then share their responses with the group, and then vote to decide which idea to pursue. PDSA cycles [37] allow change teams to rapidly test a change on a small scale, modify it in response to feedback, and test it again until the change meets the goal.

In subsequent meetings, change team members reviewed data on opioid prescribing guideline concordance and PDSA change forms prepared by the study facilitator. The change teams sought advice from the research team on matters such as how to implement treatment agreements, mental health screening, and urine drug testing. The sixth and final meeting combined a review of the clinic’s performance data, reflections from change team members, next steps for the change team, and a celebration of progress.

#### Outcome measures

This pilot test of systems consultation used the RE-AIM (Reach, Effectiveness, Adoption, Implementation, Maintenance) evaluation framework. To assess *reach*, we compared characteristics of intervention clinics, control clinics, and clinics that refused participation, including number of prescribers and characteristics of the patient panel. For *effectiveness*, we examined overall opioid prescribing rates; average morphine-equivalent daily dose for patients on long-term opioid therapy (defined as receiving an opioid prescription in each of the most recent 3 months); rates of use of treatment agreements, urine drug testing, and mental health screening; and opioid/benzodiazepine co-prescribing. Effectiveness measures used patient-level data extracted from clinics’ electronic health records. Effectiveness outcomes are reported for intervention and control clinics over the 6-month implementation period at each site by fitting least-square lines through the monthly outcome results and comparing the resulting monthly slopes and computing the *p* values of the slope estimates. We used a 12-month piecewise linear function with “knots” at 0 and 6 months, allowing for a second linear progression from months 6 to 12 to capture any continuing effect or regression to pre-intervention levels. Each clinic had equal weight in the analysis, consistent with prior organizational research conducted by the study team [27]. For *adoption*, we examined the characteristics of clinic change teams, attendance at scheduled intervention activities, and ratings by staff participants on a satisfaction survey. The survey asked a series of questions about satisfaction with the implementation strategy; responses were given on a 1 to 5 scale from strongly disagree to strongly agree. Satisfaction ratings served as a measure of acceptability. Assessment of *implementation* focused on the cost of delivering the implementation strategy. Detailed logs were kept of contact between the research team members and clinic change teams to estimate the number of hours spent delivering the implementation strategy. These hourly estimates were multiplied by an hourly rate of $140 for physician consultants (an hourly rate based on an annual salary of $195,000 for a family physician) and $38 for the facilitator. Costs for food and mileage for site visits were also included in the cost assessment. *Maintenance* was assessed using 6-month follow-up data on the effectiveness measures described above.

#### Sources of qualitative data

Qualitative data come from four sources: (1) contact logs, which were used to track all telephone calls and emails between the consulting team and clinics; (2) field notes, which were taken by members of the research team to document every meeting with clinics; (3) focus groups, which took place with each change team following their final meetings with the consultants; and (4) semi-structured debriefing interviews with the two physician consultants. Both the focus groups and interviews were conducted by a research team member who had not had previous contact with participants. Interviews and focus groups were audiotaped and transcribed verbatim. Qualitative analysis was concurrent with data collection. Members of the qualitative working group met regularly to review field notes and discuss emerging concepts and patterns, which were then described in memoranda. This paper presents qualitative findings related to formative evaluation—lessons learned from this pilot test that suggest adaptations to the intervention. (Additional qualitative results will be reported separately.) At the conclusion of the intervention period, the data (logs, field notes, memos, and focus group and interview transcripts) from all four clinics were entered into NVivo; coded using three categories: adaptation need, adaptation, and adaptation impact; and analyzed using both within-clinic and cross-clinic comparisons.

## Results

Results are organized according to the RE-AIM framework. The aim of the study corresponds to the RE-AIM domains as follows. Feasibility is assessed by one Reach measure (ease of recruitment, given under “Clinic recruitment and randomization” above) and one Implementation measure (costs, given below under “Implementation”). Acceptability is assessed by three Adoption measures (characteristics of change teams in Table 4; attendance at scheduled implementation meetings, given under “Adoption” below; and staff satisfaction ratings shown in Table 5) and two Implementation results (adaptations made to the protocol and lessons learned, both described below under “Qualitative results”). Effectiveness outcomes are shown in Table 2, and Maintenance outcomes in Table 3.

**Table 2:** Effectiveness outcomes

Through 6 months (Effectiveness)	Baseline value—control clinics	Slope of control clinics (95% CI)	*p* value (pre-post within control clinics)	Baseline value—intervention clinics	Slope of intervention clinics (95% CI)	*p* value (pre-post within intervention clinics)	Slope of intervention minus control (95% CI)	*p* value (difference between intervention—control groups)
Proportion of patients with consistent opioid Rx ^a^	0.014	− 0.0001 (0.0000, − 0.00002)	0.152	0.013	− 0.0002 (− 0.0001, − 0.0003)	0.011	− 0.0001 (0.0000, − 0.0002)	0.237
Proportion with mental health screen ^b^	0.220	0.029 (0.052, 0.006)	0.020	0.226	0.058 (0.079, 0.038)	0.009	0.029 (0.053, 0.005)	0.024
Proportion with urine drug testing ^b^	0.374	0.011 (0.035, − 0.013)	0.025	0.399	0.041 (0.061, 0.020)	0.009	0.029 (0.050, 0.008)	0.011
Proportion with treatment agreement ^b^	0.368	0.029 (0.050, 0.009)	0.009	0.428	0.059 (0.080, 0.038)	0.010	0.03 (0.051, 0.008)	0.012
Average morphine- equivalent daily dose (MEDD) ^b^	58.8	0.245 (−2.08, 2.57)	0.646	86.3	−0.337 (1.07, −1.75)	0.449	−0.581 (0.75, − 1.92)	0.425
Proportion with MEDD > 120 ^b^	0.137	− 0.002 (− 0.001, − 0.004)	0.249	0.215	−0.004 (− 0.002, − 0.005)	0.045	−0.001 (0.003, − 0.006)	0.624
Proportion with co-prescribed benzodiazepines ^b^	0.055	0.001 (− 0.006, 0.008)	0.654	0.080	− 0.001 (0.006, − 0.007)	0.637	− 0.002 (0.000, − 0.003)	0.019

^a^Three or more opioid prescriptions in each of the most recent 3 months

^b^Subset of proportion *a* above (patients with three or more opioid prescriptions in each of the most recent 3 months)

^c^*MEDD* morphine-equivalent daily dose (for patients with consistent opioid Rx)

**Table 3:** Maintenance outcomes

Through 12 months (Maintenance)	Estimated value—control clinics (6-month mark)	Slope of control clinics (95% CI)	*p* value (pre-post within control clinics)	Estimated value—intervention clinics (6-month mark)	Slope of intervention clinics (95% CI)	*p* value (pre-post within intervention clinics)	Slope of intervention minus control (95% CI)	*p* value (difference between intervention—control groups)
Proportion of patients with consistent opioid Rx ^a^	0.013	− 0.0001 (0.0000, − 0.0002)	0.007	0.012	− 0.0001 (− 0.0001, − 0.0002)	0.001	0.0000 (0.0001, − 0.0001)	0.975
Proportion with mental health screen ^b^	0.394	0.016 (0.026, 0.006)	0.002	0.574	0.033 (0.053, 0.014)	0.001	0.017 (0.028, 0.006)	0.003
Proportion with urine drug testing ^b^	0.440	0.012 (0.020, 0.005)	0.001	0.645	0.018 (0.029, 0.006)	0.002	0.005 (0.013, − 0.002)	0.153
Proportion with treatment agreement ^b^	0.542	0.031 (0.048, 0.014)	0.000	0.782	0.036 (0.056, 0.015)	0.001	0.005 (0.012, − 0.002)	0.146
Average morphine-equivalent daily dose (MEDD) ^b, c^	60.239	0.431 (0.909, − 0.048)	0.078	84.278	− 0.830 (− 0.264, − 1.396)	0.004	− 1.261 (− 0.425, − 2.097)	0.003
Proportion with MEDD > 120 ^b, c^	0.125	− 0.000 (0.002, − 0.002)	0.942	0.191	− 0.003 (− 0.001, − 0.005)	0.004	−0.003 (− 0.001, − 0.006)	0.018
Proportion with co-prescribed benzodiazepines ^b^	0.061	0.001 (0.002, − 0.001)	0.291	0.074	0.002 (0.004, − 0.001)	0.136	0.001 (0.003, − 0.001)	0.353

^a^Three or more opioid prescriptions in each of the most recent 3 months

^b^Subset of proportion *a* above (patients with three or more opioid prescriptions in each of the most recent 3 months)

^c^*MEDD* morphine-equivalent daily dose (for patients with consistent opioid Rx)

### Reach

Table 1 illustrates characteristics of clinics that agreed to participate and were assigned to receive the implementation strategy (*n* = 4), matched control clinics (*n* = 4), and clinics that were invited to participate but refused (*n* = 3). The most notable difference is that the intervention clinics were relatively large, with two of the four having patient panels over 10,000. Patient characteristics (gender, ethnicity, race) appeared relatively similar across each group of clinics.

### Effectiveness

Tables 2 and 3 contain results in the effectiveness and maintenance domains (refer to Table 2 for outcomes at 6 months and to Table 3 for outcomes at 12 months). At 6 months, statistically significant improvements were noted in the percentage of patients with mental health screening, up-to-date treatment agreements, urine drug screening, and rates of opioid-benzodiazepine co-prescribing. The overall rate of opioid prescribing and average morphine-equivalent daily dose held steady at 6 months for both intervention and control clinics.

### Adoption

Table 4 illustrates the characteristics of the clinic change teams. Each clinic change team member signed an informed consent document (we obtained a waiver of informed consent at the patient level). Attendance at scheduled implementation meetings (site visits and follow-up teleconferences/videoconferences) was 83%.

**Table 4:** Clinic change teams

Intervention clinic	Members	Composition	Attendance at intervention meetings
Team 1	6	MD, NP, RN, LPN, Lab, COM	81%
Team 2	7	MD (2), RN, MA (3), Reception	88%
Team 3	8	MD (2), RN, MA (2), Reception, Lab, COA	69%
Team 4	6	MD, RN, LPN (2), Reception, COM	92%

*Abbreviations*: *COA* Clinic Operations Assistant, *COM* Clinic Operations Manager, *LPN* Licensed Practical Nurse, *MA* Medical Assistant, *MD* Medical Doctor, *NP* Nurse Practitioner, *RN* Registered Nurse

At the end of the 6-month intervention, we administered the satisfaction survey; 24 of the 27 consented staff members responded. See Table 5 for results. Staff members’ largely positive responses indicate that participants found the intervention useful and acceptable.

**Table 5:** Clinical Staff Satisfaction Ratings

Question	Strongly agree (%)	Agree (%)	Neutral (%)	Disagree (%)	Strongly disagree (%)
I have a better understanding of the benefits and risks of long-term opioid prescribing for chronic pain	50	27	18	5	0
I am more familiar with current literature regarding evidence-based guidelines for long-term opioid prescribing for chronic pain	50	32	18	0	0
My clinic’s workflows related to opioid prescribing are easier	48	35	17	0	0
I utilize screening processes for mental health and substance abuse issues with patients who are prescribed long-term opioids for chronic pain more often	39	26	35	0	0
I utilize treatment agreements with patients who are prescribed long-term opioids for chronic pain more often	36	27	36	0	0
I utilize urine drug testing as a precautionary measure with more patients who are prescribed long-term opioids for chronic pain	32	36	32	0	0
I have more discussions with my colleagues regarding opioid prescribing for chronic pain	48	39	9	4	0
I feel more able to meet the recommendations of the ongoing UWHealth initiative related to opioid prescribing	58	38	4	0	0

### Implementation

Cost was the primary outcome in the Implementation domain. Across all four sites, the facilitator spent 237.7 h delivering the implementation strategy, which included time spent preparing for and implementing site visits, conducting email and phone correspondence with sites, and hosting monthly teleconferences/videoconferences. The two physician consultants combined to spend 85.7 h working with sites. At $140 per hour for physician consulting and $38 per hour for facilitation, respectively, consultation team costs amounted to $21,031 ($5258 per clinic) for the 6-month intervention. Providing lunches to change team members added an incentive for them to attend on-site meetings with the consultation team. Continuing medical education credits were also available to change team members. Food cost $1911.97 or $477.99 per clinic. Mileage costs for travel to site visits were $435.78. We did not attempt to monetize time spent by clinic staff members to participate in the research. We estimated that each member of the change team spent about 9 h on the study, one each for the informational session, walk-through meeting, six monthly meetings, and all-clinic teleconference. This does not include time change team members may have spent outside these study meetings to work on study-related efforts. We gave each clinic a modest stipend of $1500 to partially offset time away from clinical care required for participation in scheduled implementation activities. The total cost of delivering the intervention (i.e., consultant and facilitator time, food, mileage, and clinic stipend) was $29,379 or $7345 per clinic.

### Maintenance

Table 3 shows results at 12 months (6 months post intervention) to gauge whether clinics maintained changes in effectiveness measures. At 12 months, a statistically significant reduction in morphine-equivalent daily dose was observed in intervention clinics compared to controls (*p* = 0.003). Clinical guidelines recommend slow tapering of doses for high-risk patients to mitigate withdrawal effects. Evidently, this outcome took longer than 6 months to show significant effects in the intervention clinics. By contrast, average MEDD went up slightly in the control clinics. Intervention clinics largely maintained improvements in other outcomes, although the difference between intervention and control clinics narrowed for several outcomes showing positive results at 6 months, including urine drug testing, use of treatment agreements, and opioid-benzodiazepine co-prescribing rates (see Additional file 1). Temporal changes are evident in both intervention and control clinics, though as Tables 2 and 3 reflect, rates of change were greater in intervention clinics for various outcomes at 6 and 12 months.

### Qualitative results

The qualitative findings suggested that adaptations to the protocol were required in the following domains: clinic recruitment, composition and responsibilities of the change team, and composition and responsibilities of the consulting team. (Because the qualitative data analysis was concurrent with the intervention, the research team could make some of these modifications in real time; other adaptions await future iterations of the intervention.) Table 6 summarizes key adaptations in each domain.

**Table 6:** Adaptations/enhancements to systems consultation strategy

Intervention element	Key adaptations
Clinic recruitment	Reach out to clinic directors personally (not by email) and hold recruitment meetings in person (not by conference call). Request presence of prescribers and clinic leadership at recruitment meeting. Bring food to recruitment meeting.
Change team composition and responsibilities	Seek representation from all occupational groups and work teams affected by the intervention. Encourage participation of influential prescriber(s). Encourage change team to institute regular communication with clinic staff who are not part of the change team. Facilitate understanding of roles and responsibilities for change team members individually and collectively.
Consulting roles and responsibilities	Split consulting roles and responsibilities between a clinical expert (physician consultant) and a facilitator. Be sure that physician consultants and study facilitators are consistent in their communications to the clinic change team. Train consultants to assess clinic needs and provide tailored assistance. Clarify upfront the nature and extent of consultants’ services (e.g., not available for direct patient care). Provide explicit instruction in the purpose and use of consulting tools. Be flexible about tool use. Facilitate access to an electronic health records expert. Schedule meetings at lunchtime and provide meals. Plan and communicate agendas for meetings Support intra- and inter-clinic knowledge sharing. Leverage opportunities created by organizational policy. Recognize and make use of similarities between new opioid prescribing practices and chronic disease management protocols already in place.

Overall, these modifications may be distilled into four main lessons. First, use a personal touch to promote and sustain clinic engagement. Second, recognize the importance of clear and frequent communication: emphasize intra-clinic communication at each stage of the intervention and provide opportunities for inter-clinic communication at key points. Third, develop clear expectations for change team roles and responsibilities and explicit instruction for using implementation tools but remain flexible to accommodate the change team’s constraints and preferences. Fourth, ensure that the systems consultation team is familiar with the clinic’s organizational context and can use this knowledge to link changes to the organization’s existing workflows, policies, and values.

## Discussion

The systems consultation implementation strategy demonstrated feasibility, acceptability, and effectiveness in a study of eight primary care clinics. Clinic teams actively participated in the intervention (attendance at scheduled implementation activities was 83% of consented staff members) and reported positive feedback in focus groups and satisfaction surveys. The implementation strategy showed positive effects on several key measures of guideline concordance, including average morphine-equivalent daily dose. Opioid prescribing is a difficult issue that is receiving a great deal of scrutiny; this implementation strategy seemed to give primary care clinics the type of support they want and need to effect organizational change.

Liebschutz et al. [42] achieved positive results in an implementation trial with a similar aim of increasing opioid guideline concordance in primary care clinics. Integral to their approach was a dedicated nurse case manager who worked directly with patients on long-term opioid therapy. The systems consultation strategy includes no role for direct patient contact, instead relying on clinics’ integrating elements of the guidelines using workflow changes and delegation of tasks to existing staff. Change teams largely accomplished their goals by integrating changes within the context of standard clinical care (i.e., billable patient follow-up appointments). Evidence from both studies shows that either approach can work (case management vs. guideline integration), and both have tradeoffs. Hiring a dedicated case manager to work with chronic opioid patients can be a straightforward way to increase guideline concordance if financial resources can support the hire. Clinical staff may also prefer to delegate the work of chronic opioid management to a specialist rather than take on the responsibilities themselves. However, integrating guidelines into standards of primary care may be more feasible and sustainable in the long-term approach, especially in resource-constrained settings. Systems consultants helped clinical staff engage in potentially fraught conversations with patients on long-term opioid therapy, including the expectation of regular monitoring (via urine drug screens) and the need for regular follow-up appointments. Clinics began to use an approach for chronic pain management similar to approaches used for other chronic conditions (e.g., hypertension and diabetes) and built activities indicated by the clinical guidelines into standards of care for chronic pain (including the ability to bill for these activities).

### Limitations

The problem of opioid prescribing received attention both locally and nationally during the intervention period, and notable secular changes in opioid prescribing outcomes were evident (see Additional file 1). The UWHealth system also introduced a new opioid-prescribing policy in February 2016, concurrent with the beginning of the study period. The Centers for Disease Control and Prevention published guidelines for opioid prescribing in March 2016 that are based on the guidelines [24] used in this study. One possible interpretation is that intervention clinics were able to achieve desired changes more quickly, while control clinics started catching up on the process-related outcomes associated with the UWHealth opioids policy over time (though not on average morphine-equivalent daily dose). Notably, the UWHealth policy included a standardized treatment agreement that included all the elements of the checklist developed during this research as well as many other items. Ultimately, to avoid redundancy, the UWHealth treatment agreement replaced the checklist developed using the integrative group process. Finally, though our study design used a randomized matched-pairs design, this was not a fully powered randomized trial and the assessments of effectiveness should not be considered definitive.

## Conclusion

This research advances implementation science by demonstrating (1) a method for distilling clinical guidelines into a concise, checklist-based implementation guide and (2) a blended implementation strategy based on principles of systems engineering for successfully putting the guidelines into practice. This study showed the promise of the implementation strategy and identified improvements to it that can make it more effective for wider use. A randomized trial is being planned to use the knowledge gained during this study to deliver an adaptive implementation strategy. The planned study will more precisely reveal which elements of the implementation strategy are essential in different clinic settings, enabling us to determine the most efficient methods of promoting clinical guideline adoption for opioid prescribing in primary care.

## Additional file

Additional file 1: Figures A-G, PDF, Time series quality indicator outcomes. (PDF 350 kb)
