# Supplementary material for: A randomized matched-pairs study of feasibility, acceptability, and effectiveness of systems consultation: a novel implementation strategy for adopting clinical guidelines for Opioid prescribing in primary care
Source: Implement Sci. 2018 Jan 25;13:21. doi: 10.1186/s13012-018-0713-1 (PMC5784593; doi:10.1186/s13012-018-0713-1)
Supplement: Additional file 1: — Figures A-G, PDF, Time series quality indicator outcomes. (PDF 350 kb) [file 13012_2018_713_MOESM1_ESM.pdf]

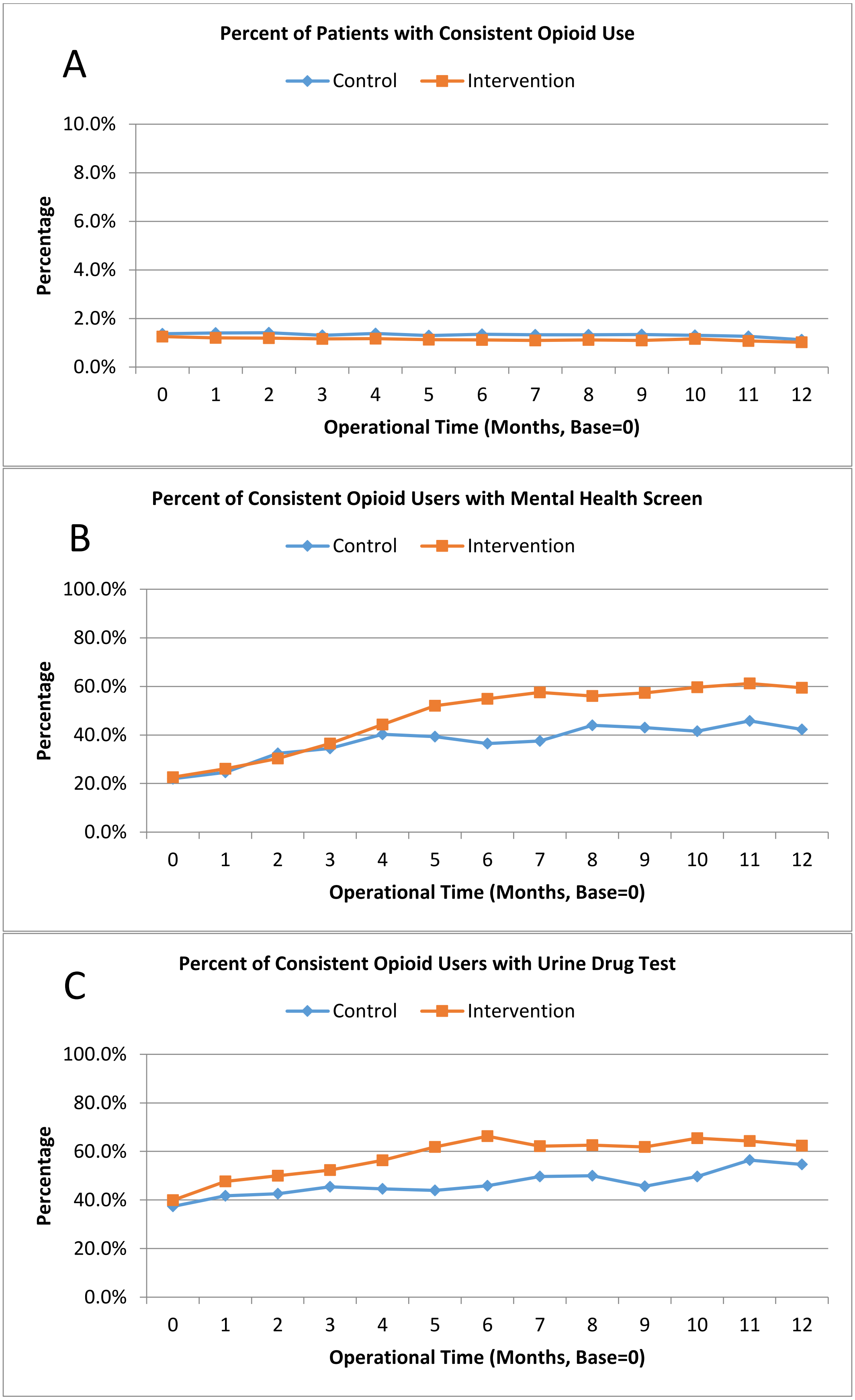

Percent of Patients with Consistent Opioid Use
A
Control
Intervention
10.0%
8.0%
6.0%
4.0%
2.0%
0.0%
Percentage
0 1 2 3 4 5 6 7 8 9 10 11 12
Operational Time (Months, Base=0)
Percent of Consistent Opioid Users with Mental Health Screen
B
Control
Intervention
100.0%
80.0%
60.0%
40.0%
20.0%
0.0%
Percentage
0 1 2 3 4 5 6 7 8 9 10 11 12
Operational Time (Months, Base=0)
Percent of Consistent Opioid Users with Urine Drug Test
C
Control
Intervention
100.0%
80.0%
60.0%
40.0%
20.0%
0.0%
Percentage
0 1 2 3 4 5 6 7 8 9 10 11 12
Operational Time (Months, Base=0)

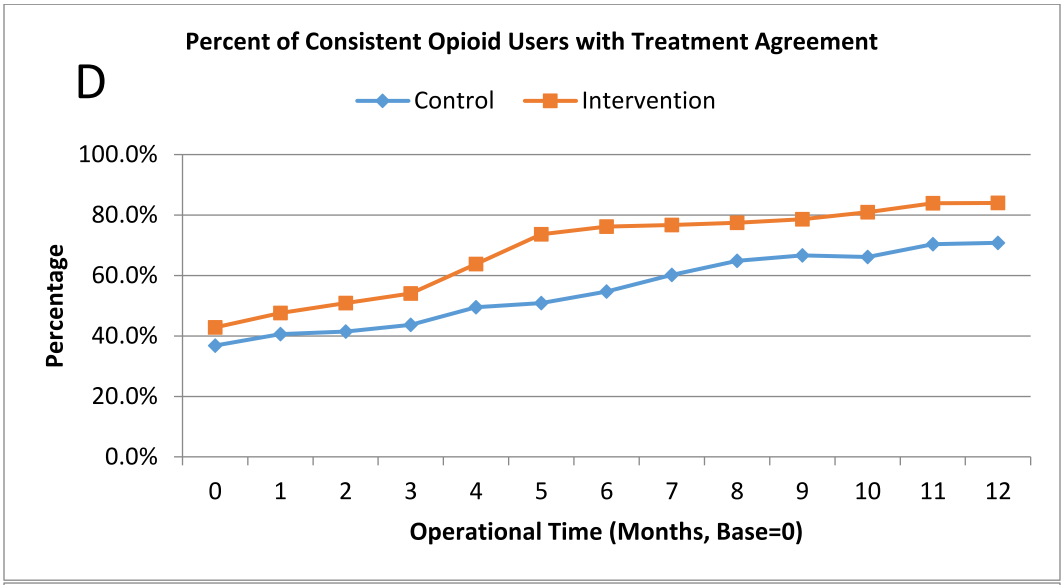

Percent of Consistent Opioid Users with Treatment Agreement
D
Control
Intervention
100.0%
80.0%
60.0%
40.0%
20.0%
0.0%
Percentage
0 1 2 3 4 5 6 7 8 9 10 11 12
Operational Time (Months, Base=0)

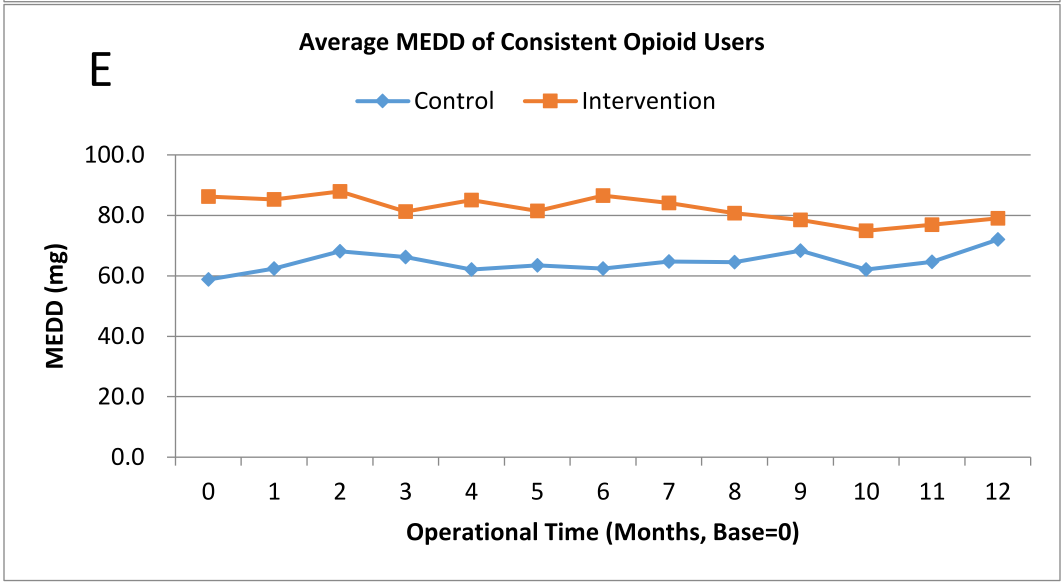

Average MEDD of Consistent Opioid Users
E
Control
Intervention
100.0
80.0
60.0
40.0
20.0
0.0
MEDD (mg)
0 1 2 3 4 5 6 7 8 9 10 11 12
Operational Time (Months, Base=0)

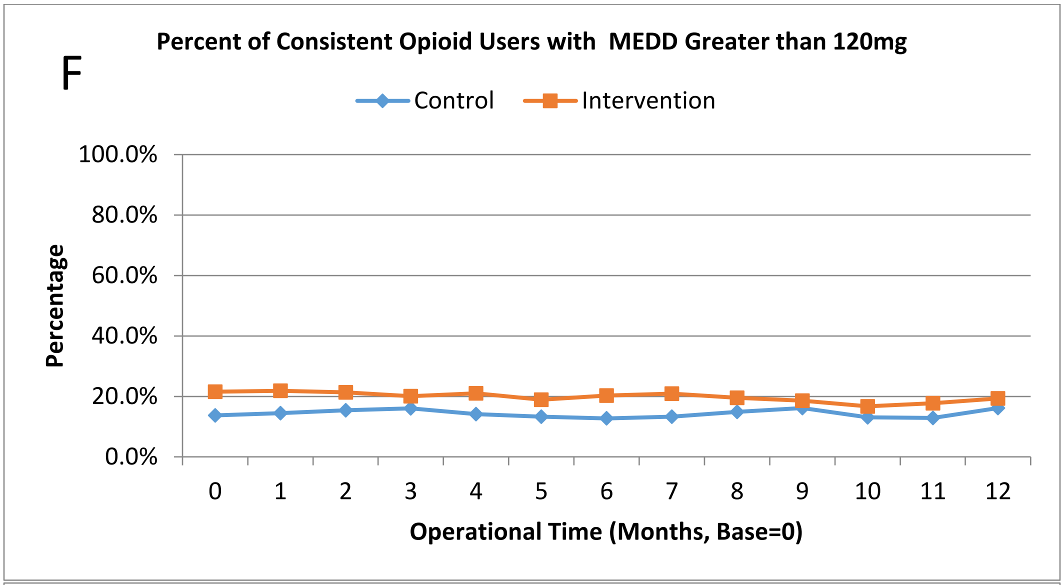

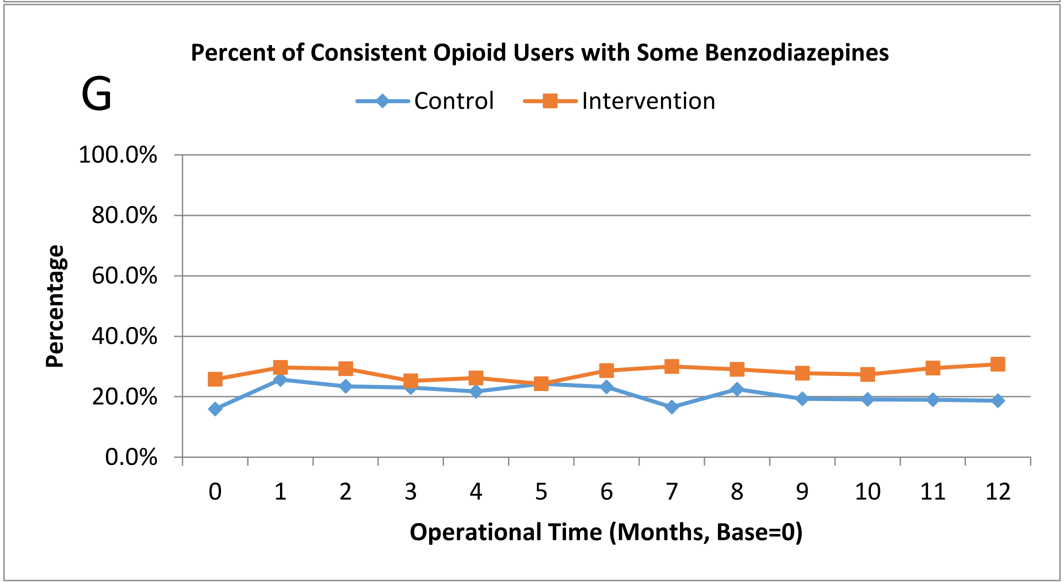

Figures A-G. Time series quality indicator outcomes
